# Microparticles for controlled growth differentiation factor 6 delivery to direct adipose stem cell‐based nucleus pulposus regeneration

**DOI:** 10.1002/term.2882

**Published:** 2019-06-19

**Authors:** Tom Hodgkinson, Jasmine Z. Stening, Lisa J. White, Kevin M. Shakesheff, Judith A. Hoyland, Stephen M. Richardson

**Affiliations:** ^1^ Division of Cell Matrix Biology and Regenerative Medicine, School of Biological Sciences, Faculty of Biology, Medicine and Health University of Manchester, Manchester Academic Health Science Centre Manchester UK; ^2^ Division of Regenerative Medicine and Cellular Therapies, School of Pharmacy, Faculty of Science University of Nottingham Nottingham UK; ^3^ Central Manchester Foundation Trust, Manchester Academic Health Science Centre NIHR Manchester Biomedical Research Centre Manchester UK

**Keywords:** adipose stem cell, growth differentiation factor 6, intervertebral disc degeneration, microparticle, nucleus pulposus, regeneration

## Abstract

Currently, there is no effective long‐term treatment for intervertebral disc (IVD) degeneration, making it an attractive candidate for regenerative therapies. Hydrogel delivery of adipose stem cells (ASCs) in combination with controlled release of bioactive molecules is a promising approach to halt IVD degeneration and promote regeneration. Growth differentiation factor 6 (GDF6) can induce ASC differentiation into anabolic nucleus pulposus (NP) cells and hence holds promise for IVD regeneration. Here, we optimised design of novel poly(DL‐lactic acid‐co‐glycolic acid) (PLGA)–polyethylene glycol–PLGA microparticles to control GDF6 delivery and investigated effect of released GDF6 on human ASCs differentiation to NP cells. Recombinant human (rh)GDF6 was loaded into microparticles and total protein and rhGDF6 release assessed. The effect of microparticle loading density on distribution and gel formation was investigated through scanning electron microscopy. ASC differentiation to NP cells was examined after 14 days in hydrogel culture by quantitative polymerase chain reaction, histological, and immunohistochemical staining in normoxic and IVD‐like hypoxic conditions. RhGDF6 microparticles were distributed throughout gels without disrupting gelation and controlled rhGDF6 release over 14 days. Released GDF6 significantly induced NP differentiation of ASCs, with expression comparable with or exceeding media supplemented rhGDF6. Microparticle‐delivered rhGDF6 also up‐regulated sulphated glycosaminoglycan and aggrecan secretion in comparison with controls. In hypoxia, microparticle‐delivered rhGDF6 continued to effectively induce NP gene expression and aggrecan production. This study demonstrates the effective encapsulation and controlled delivery of rhGDF6, which maintained its activity and induced ASC differentiation to NP cells and synthesis of an NP‐like matrix suggesting suitability of microparticles for controlled growth factor release in regenerative strategies for treatment of IVD degeneration.

## 1 INTRODUCTION

Cell‐mediated regenerative therapy, in combination with delivery of small bioactive molecules, is an attractive strategy for the treatment of intervertebral disc (IVD) degeneration, a leading cause of low back pain (Balagué, Mannion, Pellisé, & Cedraschi, 2012; Cheung et al., 2009). Low back pain affects approximately 84% of the global population, resulting in a significant socio‐economic burden surpassing many other causes of disability, including arthritis (Juniper, Le, & Mladsi, 2009; Maniadakis & Gray, 2000; Martin et al., 2008; Risbud & Shapiro, 2014). Although current conservative treatments offer relief from pain and improved functionality in the short term, progressive degeneration can make surgery necessary. However, surgical intervention often results in adjusted spinal loading, and in turn, degeneration of IVDs adjacent to intervention sites (Atlas, Keller, Wu, Deyo, & Singer, 2005). Hence, novel approaches to regenerate tissue and restore function are required.

IVD degeneration arises from cell‐mediated changes in the extracellular matrix (ECM) of the central portion of the disc, the nucleus pulposus (NP). Inflammatory positive feedback‐directed mechanisms progressively degrade the healthy Type 2 collagen and aggrecan‐rich matrix, replacing it with Type 1 collagen. This reduces osmotic pressure in the NP, altering load bearing and resulting in mechanical failure of the annulus fibrosus (AF; a fibrous collagenous tissue circumferentially enclosing the NP). Progressive injury, including tearing and fissure formation, to the AF and NP from increased localised compressive stress creates a proinflammatory environment, inflaming surrounding tissues and further increasing the expression of catabolic enzymes and factors (Johnson, Schoepflin, Choi, Shapiro, & Risbud, 2015; Risbud & Shapiro, 2014). This process is concomitant with in‐growth of blood vessels and nociceptive nerve fibres into the IVD, facilitating immune cell infiltration and thought to increase pain (Freemont et al., 1997; Hughes, Freemont, Hukins, McGregor, & Roberts, 2012).

Therapeutic regeneration strategies aim to abrogate this proinflammatory, catabolic positive feedback loop through the implantation of healthy cells and/or signalling molecules to instruct correct ECM production, particularly in the NP. One growth factor recently proposed for NP regeneration is growth differentiation factor 6 (GDF6), a member of the bone morphogenetic protein (BMP) family that plays important developmental roles in the formation of cartilaginous tissues, including the IVD (Clendenning & Mortlock, 2012; Settle et al., 2003; Tassabehji et al., 2008; Wei et al., 2016). GDF6 expression has been linked to anti‐ageing effects in multiple tissues (Hisamatsu, Ohno‐Oishi, Nakamura, Mabuchi, & Naka‐Kaneda, 2016) and, whereas studies conducted in our laboratory and others do not suggest GDF6 expression decreases with degeneration (Gulati, Chung, Wei, & Diwan, 2015; Le Maitre, Freemont, & Hoyland, 2009), preclinical studies have demonstrated that injection of GDF6 into experimentally induced rabbit and ovine annular puncture models attenuates pro‐inflammatory molecular changes and prevents progression of IVD degeneration (Miyazaki et al., 2018; Shen et al., 2009). However, the hypocellular nature of the tissue and the aberrant phenotype of degenerate NP cells suggests that growth factor therapy alone may be insufficient to promote regeneration of human IVD. We believe a therapy which combines cells and an appropriate signalling molecule, such as GDF6, may offer benefits over monotherapy approaches.

Due to difficulties in extracting and expanding autologous or allogeneic NP cells, multipotent adult stem cells, such as those derived from bone marrow or adipose tissue, have emerged as promising cell sources (Risbud et al., 2004; Rodrigues‐Pinto et al., 2016; Sakaguchi, Sekiya, Yagishita, & Muneta, 2005; Strassburg, Richardson, Freemont, & Hoyland, 2010). Recently, we demonstrated the in vitro differentiation of bone marrow‐derived mesenchymal stem cells (MSCs) and adipose stem cells (ASCs) into an NP phenotype through stimulation with a total of 700 ng recombinant human GDF6 (rhGDF6) delivered exogenously as a media supplement over a 14‐day timecourse (Clarke et al., 2014). Furthermore, stimulation with rhGDF6 produced an NP, rather than a chondrocytic phenotype, more efficiently than other factors commonly used for chondrogenic and NP differentiation of MSCs in vitro, specifically TGFβ3 and GDF5. This was evidenced by expression of defined NP‐specific marker genes, total sulphated glycosaminoglycan (sGAG) production, and importantly increased aggrecan to collagen Type 2 ratio, which constitutes an important difference between NP and articular cartilage ECM (Mwale, Roughley, & Antoniou, 2004). Significantly, ASCs were observed to adopt an NP phenotype significantly more efficiently than MSCs.

However, in order to translate a combined ASC–rhGDF6 therapy to clinic, a delivery system that controls the release of GDF6, prolonging its presence in the IVD, protecting it from rapid enzymatic degradation and lowering required effective doses is a desirable target. This is especially pertinent in IVD regeneration, where repeated injection may trigger further degeneration (Han et al., 2008; Hsieh, Hwang, Ryan, Freeman, & Kim, 2009; Zhang, La Marca, Hollister, Goldstein, & Lin, 2009). Control of growth factor delivery can be achieved by incorporating the growth factor into a biomaterial carrier system to provide protection from proteolytic enzymes and prevent growth factor degradation (Vo, Kasper, & Mikos, 2012). Spatio‐temporal control of growth factor delivery has been achieved via encapsulation within polymeric microparticles (MPs), whereby regulation of release duration has been achieved alongside limiting growth factor dose (Chen, Silva, Yuen, & Mooney, 2007; G. T. S. Kirby et al., 2011; Sands & Mooney, 2007). Homo and copolymers of lactide and glycolide have found particular application as delivery vehicles as they are FDA‐approved for various clinical functions (Middleton & Tipton, 2000), degrade in vivo into natural products (lactic and glycolic acid) that are processed by normal metabolic pathways (Göpferich, 1996; Lu et al., 2000), and have tuneable physico‐chemical properties (Vo et al., 2012). Controlled release from MPs formed from polymers such as poly(DL‐lactic acid‐co‐glycolic acid) (PLGA) is typically dependent upon the degradation rate, which in turn can be altered by changing the lactide: glycolide ratio or molecular weight (Keles, Naylor, Clegg, & Sammon, 2015). Previously, we have developed improved methods for growth factor delivery by decoupling release kinetics from polymer degradation by the inclusion of a more hydrophilic component, such as polyethylene glycol (PEG; White et al., 2013). The addition of an “in‐house” developed triblock copolymer, PLGA–PEG–PLGA provides additional control over polymer hydration to achieve sustained delivery (G. Kirby et al., 2016; White et al., 2013)

Therefore, the aim of this study was to encapsulate rhGDF6 in novel PLGA/PLGA–PEG–PLGA MPs to control its release over a defined timescale in vitro. Polymer MP delivery was investigated as this system offers high growth factor entrapment efficiency, the dry MPs have a long storage life, and MPs can be easily incorporated into injectable load‐bearing hydrogel systems without significant detriment to mechanical properties [37]. The biological effects of the released rhGDF6 on the differentiation of primary human ASCs in 3D culture were assessed and compared with rhGDF6 delivered exogenously as a media supplement.

## 2 METHODS

### 2.1 Triblock copolymer fabrication and characterisation

The triblock copolymer PLGA–PEG–PLGA was prepared in‐house and analysed using previously described methods (White et al., 2013). Briefly, synthesis occurred by ring‐opening polymerisation of the DL‐lactide and glycolide monomers in the presence of PEG and the Sn (Oct)2 catalyst under a dry nitrogen atmosphere, as reported (Hou et al., 2008; Zentner et al., 2001). The reaction was allowed to proceed for 8 hr and the resultant copolymer was dissolved and precipitated in water to remove unreacted monomers. The triblock copolymer was then dried under vacuum and stored at −20°C until required.

Characterisation of the triblock copolymer was undertaken as previously described (White et al., 2013). Nuclear magnetic resonance analysis of the copolymer was undertaken using a Bruker DPX‐300 Spectrometer (300 MHz) with deuterated choloroform (CDCl3) as the solvent. A tetramethylsilane signal was taken as the zero chemical shift. The composition of the copolymer was determined by 1H nuclear magnetic resonance by integrating the signals pertaining to each monomer, that is, peaks from CH2 of ethylene glycol and glycolide and CH and CH3 from DL‐lactide (Hou et al., 2008). The molecular weight and polydispersity index of the copolymer was determined using gel permeation chromatography (PL‐GPC 120, Polymer Labs) with differential refractometer detection. Tetrahydrofuran was employed as an eluent, with two columns (30 cm, PolarGel‐M) in series calibrated against polystyrene standards. Characteristics of the triblock copolymer are presented in Table 1.

**Table 1:** Triblock copolymer characteristics

M_N_ a	% mole lactide^a^	% mole glycolide^a^	M_N_ b	M_W_ b	PDI^b^
1600–1500–1600	75	25	3026	4582	1.51

a Determined by ^1^H nuclear magnetic resonance.

b Determined by gel permeation chromatography (GPC).

### 2.2 MP fabrication

MPs were prepared according to a previously described method involving a water/oil/water double‐emulsion solvent evaporation technique (G. T. S. Kirby et al., 2011; White et al., 2013). PLGA polymer with a lactide: glycolide ratio of 85:15 (PLGA 85:15 DLG 4CA LP421 53 kDa) was purchased from Surmodics (Eden Prairie, MN USA). Briefly, 0.7 g PLGA and 0.3 g triblock (PLGA–PEG–PLGA), that is, 1 g in total, was dissolved in dichloromethane (5 mL) to form the oil phase. A protein mixture consisting of 1 mg rhGDF6 and 9 mg human serum albumin (HSA; for rhGDF6‐MPs), or 10 mg HSA (for control‐MPs) was dissolved in deionised water (100 μL). The aqueous solution was added to the oil phase and homogenised using an LM5 axial impeller mixer (Silverson machines, Ltd, Chesham, UK) for 2 min at 4,000 rpm. This primary water/oil emulsion was quickly added to a 200 mL bath of polyvinyl alcohol (PVA) (0.3%) and homogenised again for 2 min at 9,000 rpm. This water/oil/water emulsion was stirred at 300 rpm on a Variomag 15‐way magnetic stirrer for 4 hr to facilitate DCM evaporation. MPs were then filtered, washed and lyophilized (Edwards Modulyo, IMA Edwards, UK) until dry. Control (blank) MPs were manufactured without protein, using 100 μl distilled water in the primary emulsion. Blank MPs of two different size ranges were fabricated for use in collagen gel experiments: <50 μm, denoted “small” and ~100 μm, denoted “large.” Small MPs were prepared as described above. For large particles, the aqueous solution was added to the oil phase as described above, that is, homogenised at 4,000 rpm for 2 min and the water/oil emulsion was then added to the PVA bath and homogensised for 2 min at 2000 rpm.

### 2.3 MP characterisation

MPs (50 mg/ml in double deionised water) were sized using a laser diffraction method (Coulter LS230, Beckman Coulter, UK) with agitation to prevent particles settling. To assess the morphology of the MPs, thin layers of freeze‐dried MPs were adhered to an adhesive stub and gold sputter coated for 4 min at 30 mA (Balzers SCD 030 gold sputter coater, Balzers, Liechenstein). MPs were imaged using a JSM 6060LV scanning electron microscope (SEM; JEOL, Welwyn Garden City, UK) with the accelerating voltage set to 10 kV.

### 2.4 Quantification of GDF6 release from PLGA MPs

To quantify the controlled release of total protein and GDF6 from MPs in culture conditions, micro‐bicinchoninic acid assay (BCA) assays (Pierce) and GDF6 enzyme‐linked immunosorbent assay (ELISA) assays (Life Span Biosystems) were utilised, respectively. Aliquots (25 mg) of the MPs (triplicate samples from each batch) were suspended in 1.5 ml phosphate‐buffered saline (PBS, pH 7.4); samples were gently rocked on a 3D shaker (Gyrotwister, Fisher Scientific UK Ltd) at 5 rpm in a humidified incubator at 37°C. At defined time intervals, the PBS was removed from the MPs and replaced with 1.5 ml fresh PBS; all liquid above the particles was collected without removing particles. The removed supernatants were stored frozen until required and were then assayed for total protein content using the micro‐BCA assay kit with a standard curve of HSA (0–40 μg/ml). For analysis of GDF6 release, sandwich ELISA assays were conducted according to manufacturer's recommendations (Life Span Biosystems) and GDF6 concentrations determined by comparison with defined GDF6 standards.

### 2.5 ASC isolation and culture

Subcutaneous adipose tissue was obtained from patients undergoing hip‐replacement surgery with full written informed consent, and all procedures and experiments were performed with relevant Research Ethics Committee and The University of Manchester approvals. ASCs were isolated as previously described (Burrow, Hoyland, & Richardson, 2017; Clarke et al., 2014). Briefly, adipose tissue was dissected from non‐adipose tissue, minced into small pieces and incubated at 37°C in 15 ml Hanks balanced salt solution containing 0.2% (w/v) Type 1 collagenase and 20 m*M* calcium chloride for 2 hr with gentle agitation to allow digestion of the tissue. The digested solution was filtered through a 70‐μm cell strainer, neutralised with MSC expansion medium (αMEM (Sigma‐Aldrich) containing 110 mg L^−1^ sodium pyruvate, 1000 mg L^−1^ glucose, 100 U ml^−1^ penicillin, 100 μg ml^−1^ streptomycin and 0.25 μg ml^−1^ amphotericin, 2 m*M* GlutaMAX (Life Technologies) and 10% (v/v) FBS) and centrifuged for 5 min. Finally, the supernatant was aspirated, cells resuspended in MSC expansion medium, and adherent cells cultured to confluence. Cells were incubated at 37°C with 5% CO_2_ and 20% O_2_ unless otherwise stated. The CD profile of ASCs was analysed through flow cytometry and multipotency assessed along the three mesenchymal lineages by using standard methods (data not shown; Burrow et al., 2017). Cells at Passage 3 or below were used for subsequent experiments.

### 2.6 3D collagen gel cultures with and without MPs

Type 1 collagen hydrogels were prepared to a final cell density of 4 × 10^6^ ml^−1^ (Clarke et al., 2014) by resuspending an appropriate number of pelleted ASCs in one‐part complete media prior to the addition of the eight‐parts atelosoluble Type 1 collagen (Devro, pH 2, 3 mg ml^−1^). The solution was then neutralised to pH 7 with one‐part phosphate buffer (0.2 M sodium phosphate, 1.3 M sodium chloride, pH 11), briefly mixed by pipetting to distribute the cells evenly and a volume of 100 μl was transferred into high density translucent membrane cell culture inserts (pore size 0.4 μm, BD Biosciences). If gels were to contain MPs, these were added at the correct concentration to the one‐part complete media and mixed with the collagen to disperse them within the gel. The culture inserts were maintained with 750 μl media within the well of the plate and the plates transferred to the incubator at 37°C for 1 hr before 500 μl media was pipetted on top of the hydrogel itself to ensure adequate diffusion of nutrients. After 24 hr, culture medium was replaced with differentiating media consisting of high‐glucose Dulbecco's Modified Eagles Medium (DMEM), 1% FCS, insulin‐transferring‐selenium (ITS‐X; Gibco, NY, USA), 100 μM ascorbic acid‐2‐phosphate, 1.25 mg ml^−1^ bovine serum albumin, 10^−7^ *M* dexamethasone, 5.4 μg ml^−1^ linoleic acid, 40 μg ml^−1^ L‐proline,100 U ml^−1^ penicillin, 100 μg ml^−1^ streptomycin, 2.5 μg ml^−1^ amphotericin B, and if designated, 100 ng ml^−1^ GDF 6. Media was changed every 48 hr, which included 100 ng ml^−1^ rhGDF6 in culture groups receiving exogenous rhGDF6 supplementation.

### 2.7 Hypoxic gel culture

To study the effect of hypoxia on MP‐released GDF6‐induced NP marker gene expression, ASCs were set up in 3D collagen gel culture a described above with either GDF6‐MPs or control‐MPs in hypoxic (5% O_2_) and normoxic culture conditions for 14 days. Media was changed every other day. After 14 days, RNA was extracted for gene expression analysis and immunohistochemistry staining performed.

### 2.8 Scanning electron microscopy

Acellular collagen gels were prepared as above with determined concentrations of MPs. Gels were mixed and allowed to gel at 37°C for 1 hr. Gels were subsequently submerged in media incubated at 37°C overnight. For analysis, gels were removed from well inserts, fixed in 3% gluataraldehyde, dehydrated through an ethanol gradient, and critical point dried. For cross‐sectional analysis, dry constructs were then dissected vertically through the midline and the exposed faces imaged through SEM.

### 2.9 Smad1 phosphorylation ELISA

Cells were seeded into wells at a density of 5 × 10^4^ cells per cm^2^ and serum starved for 24 hr. Cells were then washed with PBS and incubated in serum‐free MSC expansion media with and without rhGDF6. RhGDF6 from MPs was obtained through prior incubation in sterile PBS to release MP contents. This was quantified through GDF6 ELISA (as above). RhGDF6‐MP and exogenous rhGDF6 were added to relevant test cultures at 100 ng ml^−1^ and cells incubated with or without rhGDF6 for 1 hr at 37°C. After incubation, cells were washed three times with ice‐cold PBS and protein extracted with cell lysis buffer (provided with ELISA assay) supplemented with protease and phosphatase inhibitors (Life Technologies). Protein was quantified through BCA assay (Pierce) and normalised per well. Phospho‐smad1 ELISA assays (RayBiotech) were performed according to manufacturer's instructions and smad1 phosphorylation quantified through measuring absorbance at 450 nm.

### 2.10 Quantitative real‐time PCR

For RNA extraction, cells were disrupted in TRI Reagent (Sigma–Aldrich) and RNA isolated, quantified through ultraviolet spectroscopy (NanoDrop, Thermo Scientific) and cDNA synthesized as previously described (Burrow et al., 2017). To analyse the gene expression profiles of different cDNA samples, quantitative polymerase chain reaction (qPCR) reactions were undertaken using the TaqMan qPCR system. FAM‐BHQ1 probes were utilised at a concentration of 450 mM and optimal primer concentration determined empirically (Table 2). QPCR reactions were run on an Applied Biosystems StepOne Plus Real Time PCR System. The reaction master mix contained 5 μl 2X Lumino Ct qPCR Readymix, 1 μl forward primer, 1 μl reverse primer, and 0.5 μl probe and 0.5 μl 40X ROX internal reference dye. Mastermix was vortexed and 8 μl pipetted into each well of the 96‐well plate. Biological samples were examined in triplicate and 2 μl of cDNA (5 ng/μl) was pipetted into each well. A positive and negative sample was run for each gene examined to ensure no false positives; total human RNA and molecular grade H_2_O replaced cDNA, respectively. The reactions were cycled under the following conditions: 95°C for 20 s followed by 40 cycles of 95°C for 1 s and 60°C for 20 s. Data was analysed according to the 2^‐ΔCt^ method outlined by Livak and Schmittgen (Livak & Schmittgen, 2001) and normalised to two internal prevalidated reference genes MRPL19 and GAPDH.

**Table 2:** Quantitative polymerase chain reaction primer and probe sequences used in this study

Gene	Forward primer	Reverse primer	Probe (FAM‐MGB)
SOX9	GCTCTGGAGACTTCTGAA	GGTACTTGTAATCCGGGTG	TCCTTCTTGTGCTGCACGCG
COL2A1	GGCTTCCATTTCAGCTATG	CAGTGGTAGGTGATGTTC	CCAACACTGCCAACGTCCAG
ACAN	GGCTTCCACCAGTGTGAC	GTGTCTCGGATGCCATACG	TGACCAGACTGTCAGATACCCCATCCA
KRT8	CCTCATCAAGAAGGATGTG	CCTGAGGAAGTTGATCTC	CTTCCAGGCGAGACTCCAGC
KRT18	CTGCTGAACATCAAGGTC	AGGCATCACCAAGATTAAAG	CTGAGATCGCCACCTACCGC
KRT19	CCATGAGGAGGAAATCAGTA	GTCACTCAGGATCTTGGC	AATCCACCTCCACACTGACCTG
FOXF1	CCGTATCTGCACCAGAAC	TGGCGTTGAAAGAGAAGA	CCGAGCTGCAAGGCATCCCG
GAPDH	CTCCTCTGACTTCAACAG	CGTTGTCATACCAGGAAA	CACCCACTCCTCCACCTTTGA
MRPL19	CCACATTCCAGAGTTCTA	CCGAGGATTATAAAGTTCAAA	CAAATCTCGACACCTTGTCCTTCG

*Note*. Primers were added per reaction at 900 n*M* with 250 n*M* probe.

### 2.11 Histological characterisation of ECM deposition

Collagen constructs were embedded in optimal cutting temperature (OCT), snap frozen in liquid nitrogen, and cryosectioned into 6‐μm sections. Aggrecan production was investigated through immunohistochemical analysis (IHC) of gels. Briefly, gels were embedded into OCT and frozen on dry ice. Gels were cut into 8‐μm thick cross‐sections and collected onto SuperFrost Plus slides. For IHC, sections were allowed to equilibrate to room temperature, formalin fixed and blocked prior to addition of aggrecan primary antibodies (Burrow et al., 2017), and incubated at 4°C overnight. The relevant HRP‐conjugated secondary antibody was added after washing (5 × 5 min TBS with 0.1% (v/v) Tween 20; TBST) and incubated at room temperature for 1 hr. Following washing with TBST, specific staining was visualised through incubation at room temperature with 3,3'‐diaminobenzidine (DAB) Chromagen. Bright field images were acquired from three independent gel cultures and representative images depicted.

### 2.12 Statistical analysis

Statistical analysis was performed with GraphPad InStat and Prism software. For qPCR, multiple comparisons and ELISA analysis data was compared using Kruskal–Wallis with Dunn's multiple comparisons tests to assess significance differences between treatment groups and unstimulated controls, with *p* values <.05 deemed significant. Numerical and graphical results are displayed as mean ± SEM.

## 3 RESULTS

### 3.1 RhGDF6 release was controlled by encapsulation into MPs in culture conditions

**Figure 1:**
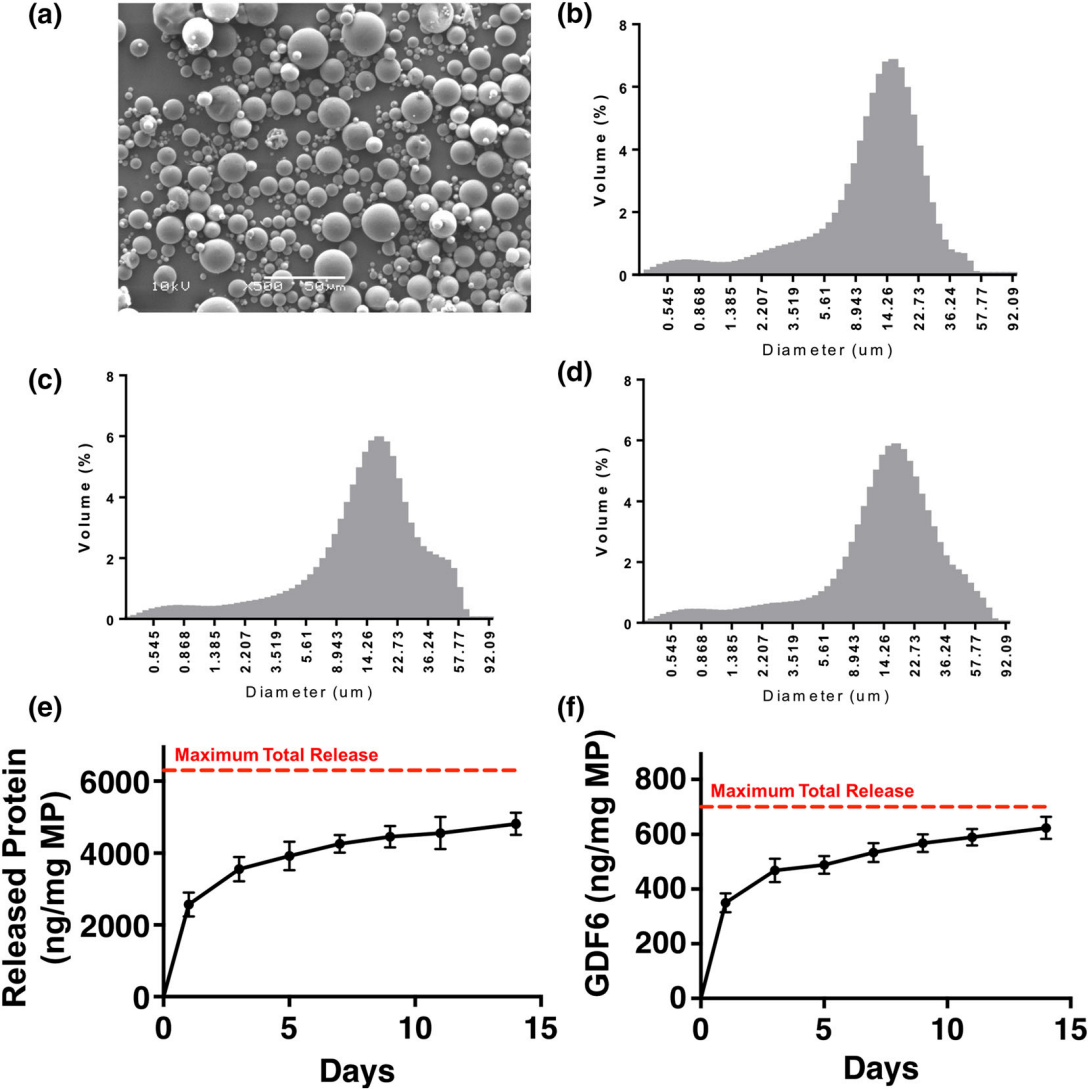
Microparticle (MP) morphology, size distribution, and protein/recombinant human growth differentiation factor 6 (rhGDF6) release in culture conditions. Size distributions and morphology of particles produced from poly(DL‐lactic acid‐co‐glycolic acid) (PLGA) 85:15 with 30% PLGA–polyethylene glycol–PLGA. (a) Representative scanning electron microscope image of particles is of microparticles produced with encapsulated human serum albumin (HSA)/rhGDF6. Size distributions of (b) HSA/rhGDF6‐encapsulated microparticles, (c) HSA only, and (d) water only control. (e) Total protein release from MPs under culture conditions was assessed over 14 days in culture through bicinchoninic acid assays on collected supernatant. Data represents means ± standard deviation (n = 3) (f) GDF6 enzyme‐linked immunosorbent assay (ELISA) analysis of rhGDF6 released from MPs over 14 days in culture conditions. Data represents means ± standard deviation (n = 3). Total protein and GDF6 ELISA results show comparable release profiles indicating controlled release over 14 days in culture [Colour figure can be viewed at wileyonlinelibrary.com]

Previous experiments demonstrated effective ASC differentiation to NP cells in 3D collagen cultures over a 14‐day culture period with a cumulative total of 700 ng rhGDF6 stimulation (Clarke et al., 2014). Therefore, MPs were designed to release their contents over this period. The MPs produced were free‐flowing, with a smooth, spherical morphology (Figure 1a). Each of the three MP formulations (‘HSA/rhGDF6’,‘HSA’, or ‘water only control’) had a similar size distribution, with mean particle sizes of 14–19 μm (Figure 1b–d). Total protein released, assessed through BCA assay, was controlled over the 14‐day culture (Figure 1e). However, an initial burst of protein, amounting to 55% of the total protein released, was released after 24 hr culture. Subsequently, 90% of the total released protein was released by 7 days culture, with the remaining 10% released by 15 days in culture. Mean total released protein was 4.75 μg/mg MP (75% of total loaded protein). The release profile was confirmed by GDF6‐specific ELISA analysis of the released protein (Figure 1f). Comparably, 56% of total delivered GDF6 was released after 1‐day culture, 85.5% by 7 days culture, and the remaining 14.5% released by 15 days culture. After 15 days, a total 620 ng/mg MP was released (88.6% of total loaded GDF6).

### 3.2 MPs could be incorporated into collagen gel 3D culture

**Figure 2:**
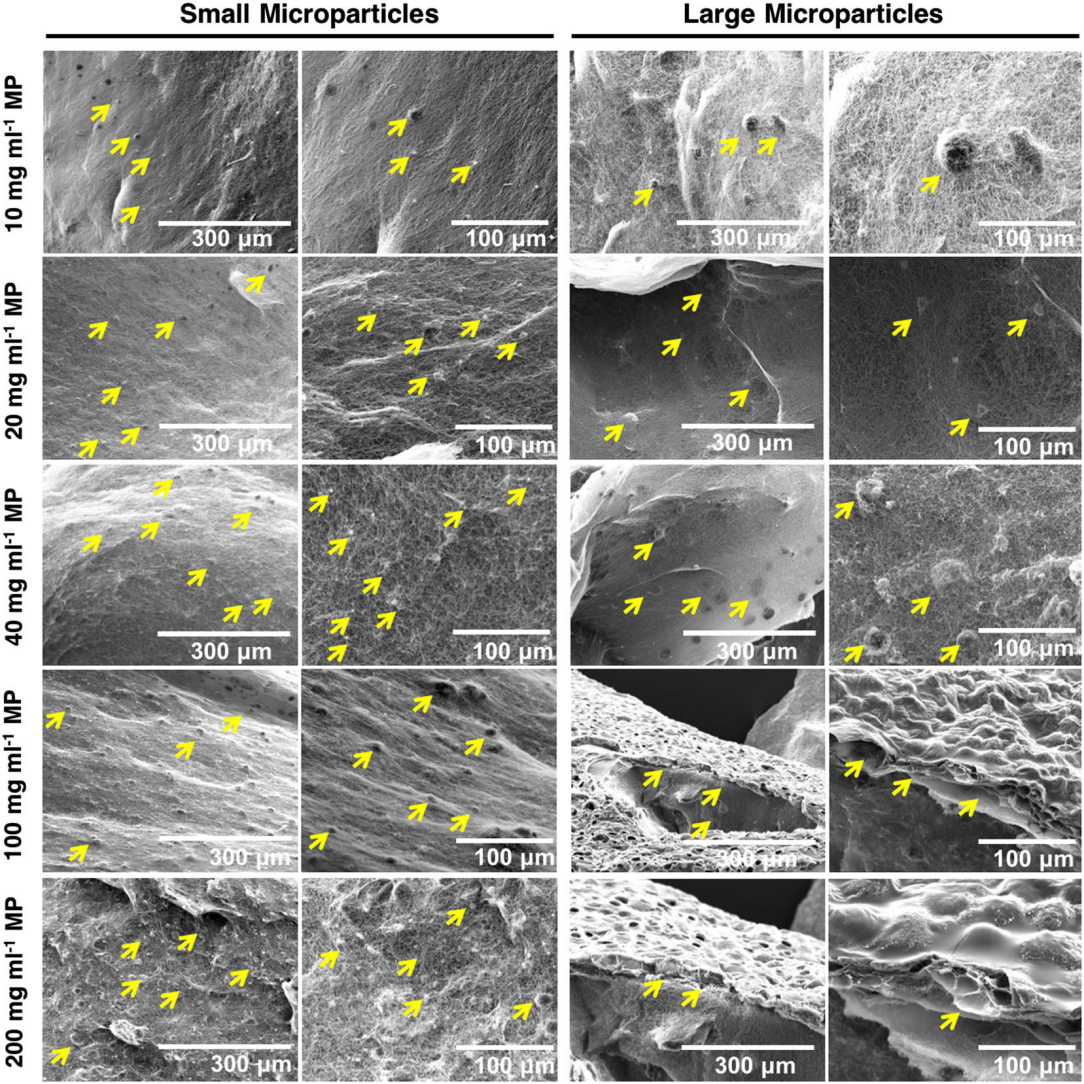
Loading density of microparticles (MPs) in collagen gels. Scanning electron microscope images of MP‐loaded collagen gels. Small microparticles (14–19 μm) demonstrated effective incorporation into collagen gels even at high density 20 times that required for effective recombinant human growth differentiation factor 6 delivery. Large MPs (100 μm) were seen to aggregate at high density and form external layers, therefore small MPs were selected for downstream experimentation [Colour figure can be viewed at wileyonlinelibrary.com]

Protein release data demonstrated that 1.13 mg of MPs (11.3 mg ml^−1^) were required per gel construct to deliver optimal (700 ng) quantities of rhGDF6 to induce human ASC differentiation to NP cells. Therefore, the effects of incorporating therapeutically relevant quantities of MPs into the collagen gel constructs were analysed. SEM confirmed effective incorporation and distribution of MPs through collagen gel constructs, even for concentrations ~20× (200 mg ml^−1^) that required for rhGDF6 delivery (Figure 2). Conversely, at therapeutic concentrations (≥10 mg ml^−1^), larger MPs (~100 μm) aggregated at gel exteriors and disrupted gel formation. This demonstrated the importance of considering MP size and hence smaller (mean 14‐19 μm) MPs were taken forward to analyse the activity of released rhGDF6.

### 3.3 RhGDF6 released from MPs increased expression of NP marker genes in ASCs

**Figure 3:**
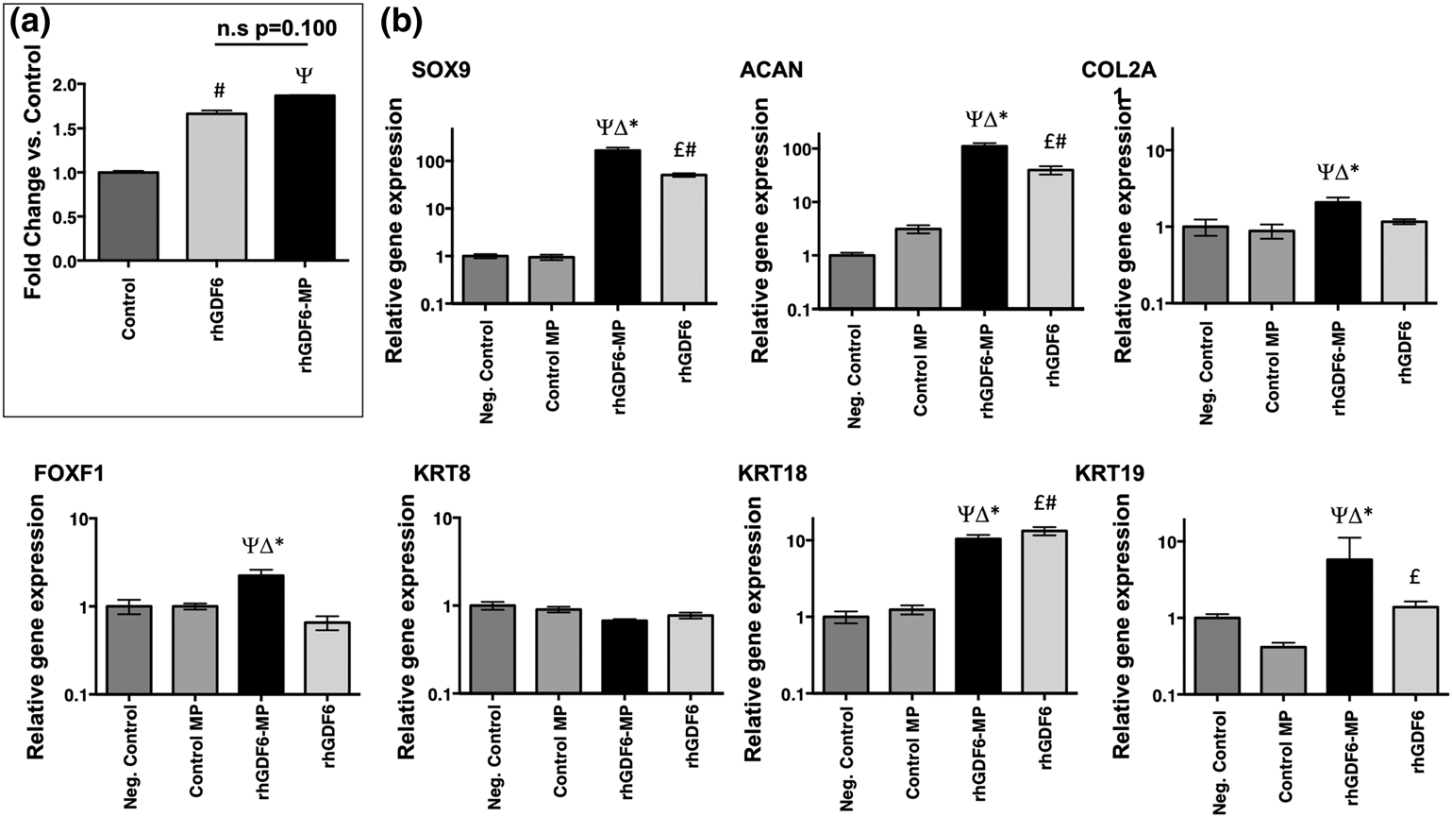
Effect of microparticle (MP)–encapsulated recombinant human growth differentiation factor 6 (rhGDF6) on SMAD response and nucleus pulposus (NP)–marker gene expression in adipose stem cells (ASCs). (a) Smad1 phosphorylation assessed by enzyme‐linked immunosorbent assay. MP‐encapsulated rhGDF6 remained active on release and was able to induce smad1 phosphorylation in ASCs with a similar effectiveness to exogenous rhGDF6 added directly to culture media. Data represents means ± standard deviation, normalised to unstimulated controls (n = 3; # p < .05 rhGDF6 vs. Control; Ψ p < .05 rhGDF6‐MP vs. Control). (b) Quantitative polymerase chain reaction analysis of NP marker gene expression after 14‐day culture. RhGDF6 delivered through MP release significantly upregulated expression of NP‐marker genes in culture. Expression was normalised to housekeeping genes (GAPDH and MRPL19) and unstimulated negative control samples. In exogenous rhGDF6 supplemented groups, 100 ng ml^−1^ rhGDF6 was added to media at each media change. Data represents means ± scanning electron microscope (n = 3; * p < .05 rhGDF6‐MP vs. rhGDF6; Δ p < .05 rhGDF6‐MP vs. Control MP; Ψ p < .05 rhGDF6‐MP vs. Negative Control; £ p < .05 rhGDF6 vs. Control MP; # p < .05 rhGDF6 vs. Negative Control)

To assess whether released rhGDF6 was active and able to activate cellular signalling pathways, phospho‐smad1 ELISA assays were performed. Smad1 phosphorylation was seen to be significantly increased in comparison with unstimulated cells (Figure 3a; *p* = .015). Smad1 activation by rhGDF6 released from MPs was comparable with rhGDF6 supplemented to media (*p* = .100) at the same concentration indicating no loss of function through encapsulation.

After 14 days, culture with rhGDF6‐MPs induced the upregulation of conventional and novel NP marker genes in comparison with unstimulated controls (Figure 3b). Conventional markers SOX9 (*p* < .0001), COL2A (*p* = .017), and ACAN (*p* < .0001) were significantly upregulated in constructs containing rhGDF6‐MPs versus unstimulated control constructs after 14 days. Additionally, there were significant increases in the novel NP marker genes FOXF1 (*p* = .013), KRT18 (*p* = .0002), and KRT19 (*p* = .027) in constructs containing rhGDF6‐MPs versus unstimulated control constructs after 14 days. Control MPs did not promote increased expression of any of the genes studied, confirming that differentiation was driven by GDF6 rather than the MPs themselves.

Crucially, MP delivery of rhGDF6 either matched or increased gene NP marker gene expression in comparison with exogenous addition of rhGDF6 to the media, with significant upregulations observed in SOX9 (*p* < .0001), COL2A1 (*p* = .0028), ACAN (*p* = .0004), FOXF1 (*p* = .0019), and KRT19 (*p* = .01) compared with exogenous rhGDF6 addition.

### 3.4 RhGDF6 released from MPs increases sGAG production by ASCs

**Figure 4:**
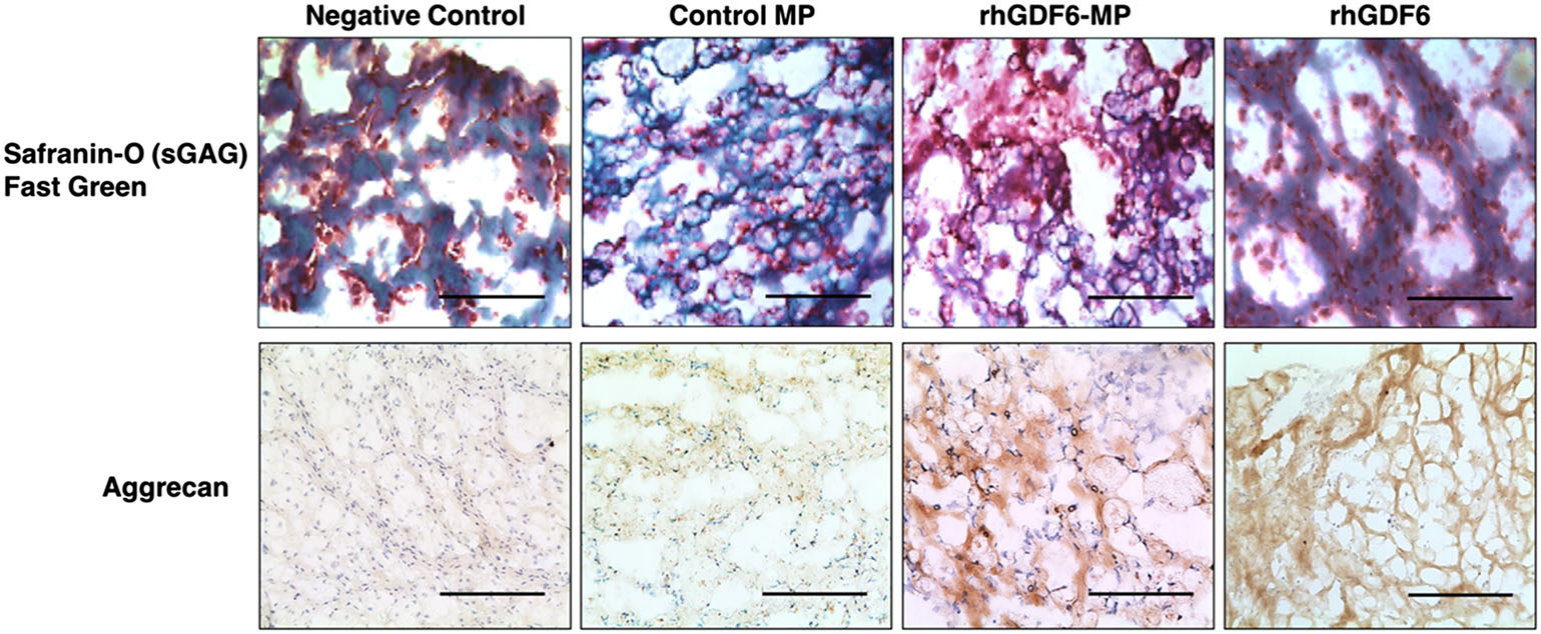
Histological and immunohistochemical staining of gel cultures after 14 days. Safranin O/Fast Green staining of gel cross‐sections after 14 days culture showing sGAG production. Greater density and distribution of sGAG staining (red) is seen in recombinant human growth differentiation factor 6 (rhGDF6)–microparticle (MP) gels in comparison with control and rhGDF6 groups. Aggrecan immunohistochemical analysis showed corresponding levels of aggrecan in test groups, with rhGDF6‐MP gels demonstrating aggrecan production and distribution through the gel. Representative images, scale bars = 300 μm [Colour figure can be viewed at wileyonlinelibrary.com]

After observing significant increases in NP‐marker gene expression in ASCs with rhGDF6‐MP stimulation, the effect of stimulation on sGAG and aggrecan synthesis and deposition by cells after 14 days in collagen gel cultures were assessed through Safranin‐O/Fast Green staining and aggrecan IHC, respectively (Figure 4). Through Safranin‐O staining, rhGDF6‐MP stimulation was shown to induce greatest sGAG staining (red) in comparison with other test groups. Analysis of gels in which rhGDF6 was delivered as a media supplement showed sGAG staining though less extensive than rhGDF6‐MP gels and restricted to pericellular areas. In control‐MP and negative control groups, limited sGAG staining was observed with larger amounts of Fast Green staining of collagenous matrix. These results were mirrored in analysis of aggrecan‐specific IHC of gels where rhGDF6‐MP–containing groups showed significantly increased aggrecan staining in comparison with negative control and control‐MP groups. RhGDF6 groups also showed increased aggrecan staining to a similar extent to MP‐delivered rhGDF6.

### 3.5 RhGDF6‐MPs maintains effective NP differentiation of ASCs in hypoxic environments

**Figure 5:**
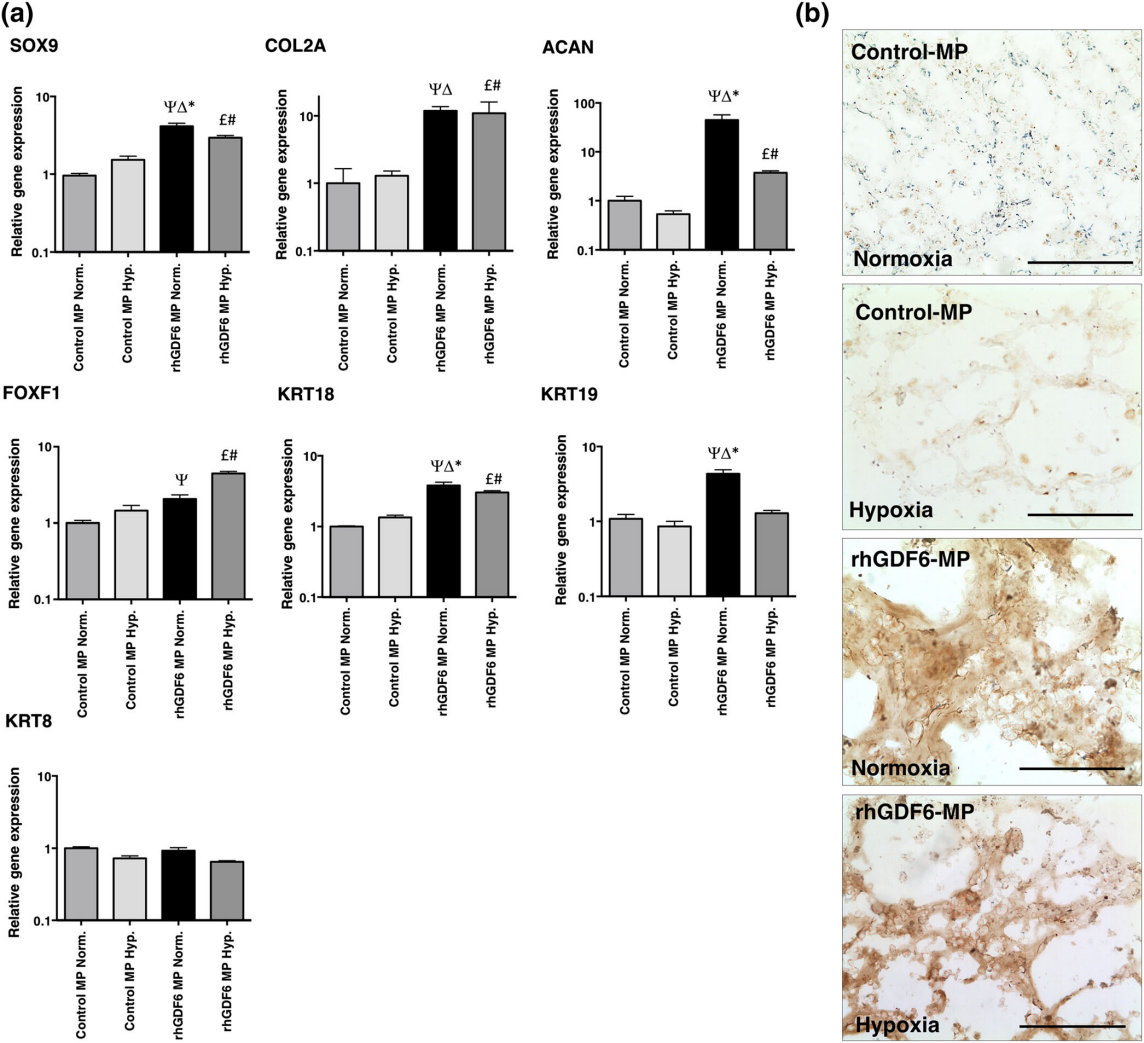
Effect of hypoxic culture conditions on effectiveness of recombinant human growth differentiation factor 6 (rhGDF6)–microparticle (MP) to induce adipose stem cell differentiation and aggrecan synthesis. (a) Quantitative polymerase chain reaction analysis of nucleus pulposus (NP)–marker gene expression after 14 days culture in normoxic (20% [v/v] O_2_) or hypoxic (5% (v/v) O_2_) conditions in the presence of rhGDF6‐MPs or control‐MPs. Expression was normalised to housekeeping genes (GAPDH and MRPL19) and unstimulated control samples. Data represents means ± scanning electron microscope (n = 3; * p < .05 rhGDF6‐MP Normoxia vs. rhGDF6‐MP Hypoxia; Δ p < .05 rhGDF6‐MP Normoxia vs. Control MP Hypoxia; Ψ p < 0.05 rhGDF6‐MP Normoxia vs. Control MP Normoxia; £ p < .05 rhGDF6‐MP Hypoxia vs. Control MP Hypoxia; # p < .05 rhGDF6‐MP Hypoxia vs. Control MP Normoxia). RhGDF6‐MPs were able to upregulate NP‐marker gene expression in hypoxic culture conditions, though in the majority of cases, no additive effect was observed through hypoxic culture. (b) Aggrecan immunohistochemical analysis staining of gel cultures after 14 days demonstrating increased deposition in normoxic versis hypoxic cultures, with enhanced staining in rhGDF6‐MP cultures compared with control cultures [Colour figure can be viewed at wileyonlinelibrary.com]

The environment of the degenerative IVD is hypoxic, and any successful regenerative therapy must function in this environment. Therefore, the effectiveness of rhGD6‐MP to induce NP gene expression in ASCs in hypoxic culture conditions was investigated and compared with normoxic culture and control MP (Figure 5a). Under normoxic conditions, the induction of NP gene expression by rhGDF6‐MP stimulation was consistent with previous experiments (Clarke et al., 2014). In hypoxic conditions, compared with hypoxic and normoxic culture with control‐MPs, rhGDF6‐MPs significantly increased the expression of SOX9 (*p* = 0.0002; *p* = .0003), COL2A (*p* = .012; *p* = .0027), ACAN (*p* = .0006; *p* = .0003), FOXF1 (*p* < 0.0001; *p* < .0001), and KRT18 (*p* = .0004; *p* = .0004). Hypoxic conditions did result in differences in expression in rhGDF6‐MP groups. Statistically significant increases in FOXF1 expression (×4.5 vs. ×2.2; *p* < .0001) were observed with hypoxic conditions but of note statistically significant decreases were observed in KRT19 (×4.3 vs. ×1.2; *p* = .0005) and ACAN (×18.6 vs. /×5.9; *p* = .009), though the latter remained significantly upregulated vs. control conditions.

Secretion of the important NP ECM molecule aggrecan by ASCs after 14 days culture was assessed using IHC (Figure 5b). Consistent with previous results, culture with rhGDF6‐MP in normoxic conditions produced an aggrecan‐rich ECM, with substantial increases in aggrecan synthesis also demonstrated in rhGDF6‐MP samples under hypoxic conditions.

## 4 DISCUSSION

Due to its anatomical location and the characteristics of the pathological progression of IVD degeneration, it is an ideal target for combined small molecule‐cell therapeutic regenerative medicine strategies. As proven in other in vivo studies utilising small molecule bioactive factors problems exist in delivered molecule half‐life, need for significantly supraphysiological doses, off‐target effects, and the need for repeated delivery, with the latter particularly problematic as needle puncture of the AF induces IVD degeneration (Han et al., 2008; Zhang et al., 2009). Therefore, the controlled release of growth factors from protective nano‐ and micro‐scale carriers offers an attractive alternative.

Here, we investigated the potential of polymer MPs to deliver rhGDF6 in a 3D hydrogel culture model to induce NP differentiation (defined by NP marker gene expression) in ASCs. Protein release data and GDF6 ELISA assays demonstrated that, as designed, encapsulated rhGDF6 delivery was controlled over 14 days under culture conditions. The MP system utilised here was designed to deliver rhGDF6 over a 14‐day period as previous work had found this to be sufficient to induce NP gene expression in ASCs (Clarke et al., 2014). However, the MPs have been developed to allow easy manipulation of both rhGDF6 “payload” and delivery time through alteration of polymer composition, which is likely to be useful in vivo for the tuning of treatments to patient needs. Furthermore, the MPs used here (mean diameter 14–19 μm) do not interfere with hydrogel gelation, even at concentrations 20 times those required to deliver optimised concentrations of rhGDF6 for in vitro differentiation. This gives significant flexibility for alteration of dose and MP density if in vivo optimisation is required. Adding this to their ease of incorporation into pre‐gel solutions prior to gelation makes this method of rhGDF6 delivery attractive as an injectable therapy for IVD degeneration.

In this system, the released rhGDF6 remained active and was able to activate smad1/5/8 signalling and significantly increased the expression of NP marker genes over 14‐days culture. The induction of NP‐marker gene expression in ASCs through rhGDF6 stimulation here corresponds with previous work (Clarke et al., 2014). Crucially, MP delivery of rhGDF6 significantly increased NP marker gene expression in comparison with rhGDF6 delivered as a media supplement in the majority of cases. This is likely due to MP delivery increasing rhGDF6 availability to cells in comparison with diffusion into gels from media. This finding is highly pertinent to IVD regeneration as the disc is an avascular tissue that relies solely on diffusion for nutrient and growth factor delivery and the fibrotic changes associated with degeneration limit such diffusion through the tissue. It also demonstrates that rhGDF6 encapsulation in MPs retained its bioactivity over the 14‐day time course, highlighting that these MPs have potential for delivery of rhGDF6 within the degenerative IVD over extended time periods without loss of bioactivity.

The degenerative NP is a hypoxic environment, therefore we tested the performance of MP delivered rhGDF6 in a 3D hypoxic culture model. In this system, culture in hypoxia did not significantly affect the expression of the majority of NP marker genes tested. Crucially, three major indicators of NP differentiation SOX9, COL2A1, and ACAN all remained statistically significantly upregulated versus unstimulated control cultures. Expression of the novel NP target KRT19 was reduced in hypoxic conditions compared with normoxic rhGDF6‐MP populations, though Keratin 18 was elevated comparably in both hypoxia and normoxia. Future research into the details of rhGDF6 signalling could be critical for fully understanding the reasons behind this effect. Aggrecan immunostaining of normoxic and hypoxic gels demonstrated that rhGDF6‐mediated stimulation of proteoglycan production is maintained even in conditions mimicking the degenerative NP suggesting that hypoxia would not be detrimental to GDF6‐induced differentiation of ASCs to NP cells and appropriate NP‐like ECM secretion.

For future translation, it would be essential to investigate the influence of total GDF6 dose, release rate, and release duration on ASC differentiation and tissue regeneration in vivo. It will also be important to assess other microenvironmental factors, including pH which is known to decrease with degeneration and has been shown to influence both native NP cell phenotype (Gilbert, Hodson, Baird, Richardson, & Hoyland, 2016) and the regenerative potential of implanted MSCs/ASCs (Naqvi & Buckley, 2016). Although degradation of PLGA/PLA‐containing MPs results in acidic by‐products, the relatively slow degradation rate of our MP system, combined with the ability to minimise MP density through optimisation of GDF6 loading and release rate, will enable clearance from the tissue through normal metabolic processes and reduce the risk of by‐product accumulation and associated reduction in pH.

## 5 CONCLUSION

In conclusion, this work has demonstrated the controlled delivery of rhGDF6 to ASCs in a 3D in vitro hydrogel model through MP encapsulation. The rhGDF6 delivered in this manner induced ASCs differentiation to NP cells and production of an aggrecan‐rich NP‐like ECM over a 14‐day period to levels at least equivalent to that achieved by exogenous addition of GDF6. Encapsulation in MPs retained rhGDF6 bioactivity over the 14‐day time‐course, and this bioactivity remained effective even under hypoxic culture conditions that mimic the degenerative NP. As such, this data serves as important proof of principle study that the MP delivery of rhGDF6 described in this manuscript offers a versatile and attractive method for future in vivo treatment of IVD degeneration and requires further investigation in vivo in appropriate degenerative IVD animal models.

## CONFLICTS OF INTEREST

The authors declare no conflicts of interest.
